# SEC-SANS: size exclusion chromatography combined *in situ* with small-angle neutron scattering^1^

**DOI:** 10.1107/S1600576716016514

**Published:** 2016-11-02

**Authors:** Ashley Jordan, Mark Jacques, Catherine Merrick, Juliette Devos, V. Trevor Forsyth, Lionel Porcar, Anne Martel

**Affiliations:** a Institut Laue–Langevin, 71 avenue des Martyrs, 38042 Grenoble, France; b Faculty of Natural Sciences, Keele University, Keele, Staffordshire ST5 5BG, UK

**Keywords:** SEC-SANS, size exclusion chromatography, small-angle neutron scattering, *in situ*, sample environment

## Abstract

The first implementation and use of an *in situ* size exclusion chromatography (SEC) system on a small-angle neutron scattering instrument (SANS) is described. The possibility of deploying such a system for biological solution scattering at the Institut Laue–Langevin (ILL) has arisen from the fact that current day SANS instruments at ILL now allow datasets to be acquired using small sample volumes with exposure times that are often shorter than a minute. This capability is of particular importance for the study of unstable biological macromolecules where aggregation or denaturation issues are a major problem. The first use of SEC-SANS on ILL’s instrument D22 is described for a variety of proteins including one particularly aggregation-prone system.

## Abstract 2

This publication describes the combination of size exclusion chromatography with small-angle neutron scattering *in situ*. The need for this technique and the progress achieved thanks to it are illustrated by relevant examples.

## 1. Introduction   

Small-angle scattering (SAS) is a powerful technique that can yield important low-resolution structural information on macromolecular systems. The X-ray and neutron scattering analogues of the technique (SAXS and SANS, respectively) are strongly complementary and provide different structural information. SAXS methods have become increasingly widely used at synchrotron radiation (SR) sources, often supporting structural information from crystallography, electron microscopy and NMR (Appolaire *et al.*, 2015 ▸; Delaforge *et al.*, 2015 ▸; Hennig *et al.*, 2014 ▸; Lapinaite *et al.*, 2013 ▸). SAXS studies typically provide overall ‘envelope’ information on protein structure, and the high fluxes available at SR sources may be exploited in parametric studies. SANS methods provide the same type of information but – crucially – offer the ability to use solvent contrast variation to distinguish and model different parts of a multi-component system, either through the availability of natural contrast (*e.g*. nucleic acid/protein complexes) or by selective deuteration (*e.g.* protein–protein complexes). SANS data collection is also effectively free of the effects of radiation damage – something that is often a major problem in SAXS work. The growth in the use of SAXS methods by structural biologists over the past 20 years or so has occurred as a result of developments in instrumentation at SR sources, as well as the availability of user-friendly analysis software. There has also been greatly increased use of SANS methods in structural biology in the recent past, with major developments in detector technology, beamline fluxes and sample provision having a strong impact on the uptake of the technique. The high demand for SAS capabilities has meant that a number of central facilities operate dedicated instruments for biological SAXS (Pernot *et al.*, 2013 ▸; Tsutakawa *et al.*, 2007 ▸; Tsuruta *et al.*, 1998 ▸) and SANS (Heller *et al.*, 2014 ▸). These accommodate efficient sample managing systems that allow high-throughput data collection (Round *et al.*, 2015 ▸; Martel *et al.*, 2012 ▸; Hura *et al.*, 2009 ▸). One of the more recent developments for SAXS has been the availability of *in situ* size exclusion chromatography (David & Pérez, 2009 ▸; Round *et al.*, 2013 ▸) (SEC), which allows SAXS data to be collected from freshly purified sample material.

In this paper we describe the first SANS measurements carried out in combination with an *in situ* SEC system on instrument D22 at the Institut Laue–Langevin (ILL). This development has only been possible because the high neutron flux on this instrument allows short exposure times on relatively small sample volumes. The new system allows data quality to be significantly improved, particularly for difficult aggregation-prone systems.

## 2. Materials and methods   

### 2.1. Sample preparation   

Gel filtration standards were purchased from BioRad, reference 151-190. This contained 5 mg of bovine thyro­globulin (670 kDa), 5 mg of bovine γ-globulin (158 kDa), 5 mg of chicken ovalbumin (44 kDa), 2.5 mg of horse myoglobin (17 kDa) and 0.5 mg of vitamin B (1.35 kDa). Each standard mixture is diluted in its elution buffer. The protonated and deuterated proteins Sir2a (34 kDa) and Alba3 (13 kDa) were prepared at the Deuteration Laboratory in the Life Sciences Group of ILL (Haertlein *et al.*, 2016 ▸) according to the protocols given below.

For recombinant protein expression of Sir2a and Alba3, pET-28a Sir2a and Alba3, clones were obtained by cloning into the pET-28a plasmid two codon-optimized gene sequences optimized from the *Plasmodium falciparum* Sir2a (Zhu *et al.*, 2012 ▸) and Alba3 genes (PF3D7_1328800 and PF3D7_1006200). These clones were transformed into the *Escherichia coli* BL21 DE3 strain. Bacterial cultures were grown in lysogeny broth medium at 310 K until an optical density at 600 nm wavelength (OD_600_) of 0.6–0.8 was reached. Both sets of cultures were induced with 1 m*M* isopropyl β-d-1-thiogalactopyranoside (IPTG) and grown for a further 3 h at 310 K and 20 h at 293 K for Sir2a and Alba3, respectively. Cells were harvested by centrifugation and stored at 193 K prior to lysis.

For deuterated Alba3 production, bacterial cultures were adapted to growth in 85% deuterated minimal media prior to inoculation of 1.5 l of fermentation culture. The fermenter culture was allowed to reach an OD_600_ of 17.0 before induction with 1 m*M* IPTG at 298 K. The culture was grown to a final OD_600_ of 21 after 23 h. Cells were harvested by centrifugation and stored at 193 K.

For purification, Sir2a frozen cell pellets were re-suspended on ice in lysis buffer (25 m*M* Tris, 300 m*M* sodium chloride, 2 m*M* imidazole pH 7.5) supplemented with benzonase nuclease and EDTA free protease inhibitor cocktail (Roche) and lysed by passing cells through a cell-disruptor three times. The cell lysates were centrifuged at 20 000 r min^−1^ for 30 min at 277 K and loaded onto a GE HiTrap TALON column using a 1 ml min^−1^ flow rate at 277 K. The column was washed with wash buffer (25 m*M* Tris, 300 m*M* sodium chloride, 20 m*M* imidazole pH 7.5) and protein eluted in 25 m*M* Tris, 300 m*M* sodium chloride, 300 m*M* imidazole pH 7.5.

Protonated and deuterated Alba3 cell pellets were re-suspended on ice in lysis buffer (50 m*M* sodium phosphate, 500 m*M* sodium chloride, 10 m*M* imidazole pH 7.0) supplemented with benzonase nuclease and EDTA free protease inhibitor cocktail and were lysed by sonication using a Sonics Vibra cell VC750 sonicator (10 s on, 59 s off, 10 cycles 50% amplitude). The cell lysates were centrifuged at 20 000 r min^−1^ for 30 min at 277 K and loaded onto a GE HisTrap Ni-NTA column using a 5 ml min^−1^ flow rate at 277 K. The column was washed with a solution of 50 m*M* sodium phosphate, 1 *M* sodium chloride, 40 m*M* imidazole pH 7.0, and protein eluted in 50 m*M* sodium phosphate, 500 m*M* sodium chloride, 500 m*M* imidazole pH 7.0.

Proteins were concentrated using Millipore 10 and 3 kDa centrifugal filtration units for Sir2a and Alba3 respectively, before gel filtration either on the SANS instrument or in a cold room right before the SANS measurement.

### 2.2. SANS data recording and reduction   

**Figure 1:**
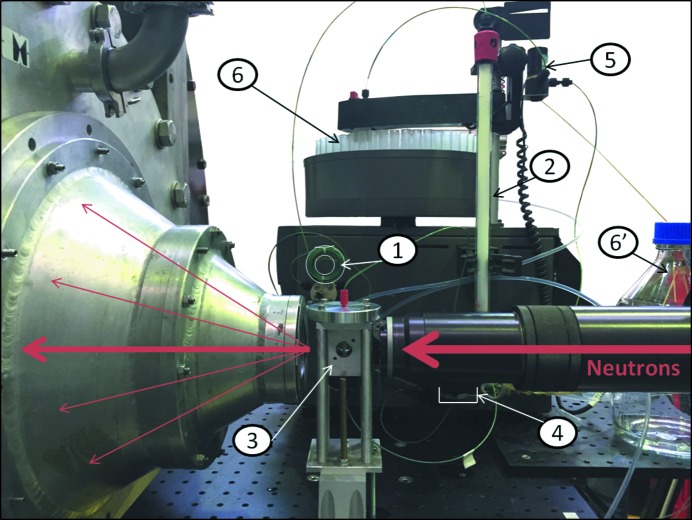
Setup of *in situ* chromatography SANS. The sample is manually injected into the loop (1), then it passes through the size exclusion column (2), the SANS sample cell (3), the UV–visible spectrophotometer (4) and the valve (5), which directs it either to the fraction collector (6) or to the waste container (6′).

SANS data were recorded on diffractometers D11 (static measurement of freshly purified sample) and D22 (*in situ* chromatography) of ILL. The *in situ* size exclusion chromatography was performed using an AKTAPrime system (GE Healthcare) with a Superdex 75 Column 10/300 GL. The samples were manually injected and then sequentially passed through the size exclusion column, the spectrophotometer measurement cell and the SANS measurement cell before being collected in a fraction collector (Fig. 1 ▸). The flow rate was maintained at 0.3 ml min^−1^ throughout data collection.

### 2.3. SANS instrument configurations   

SEC-SANS measurements on instrument D22 were carried out using a neutron wavelength of 6 Å ± 10%, detector distances of 8, 4 or 2 m, a rectangular collimation system of 55 × 40 mm having the same length as the sample–detector distance, and a rectangular sample aperture of 7 × 10 mm. The sample holder was a Suprasil quartz cell of 1 mm sample thickness placed in the ‘stopped-flow head’ (Grillo, 2009 ▸). The exposure time was 30 s. Sample injection started at the same time as SANS data acquisition. The whole setup was maintained at a temperature of 288 K.

In both experiments, the data were reduced using the *GRASP* software (https://www.ill.eu/instruments-support/instruments-groups/groups/lss/grasp/home/) and analyzed using *Igor NCNR* SANS reduction macros (Kline, 2006 ▸). The corrections applied included subtraction of the blocked beam and the empty cell scattering, transmission and thickness scaling, absolute intensity calibration using the direct beam intensity, and buffer subtraction. For SEC-SANS, the transmission of the buffer, recorded during elution of the column dead volume, was used for scaling the protein signal. Short exposures were averaged, after normalization for sample concentration, to increase the signal-to-noise ratio as necessary.

## 3. Results   

### 3.1. Proof of concept: calibration standards   

**Figure 2:**
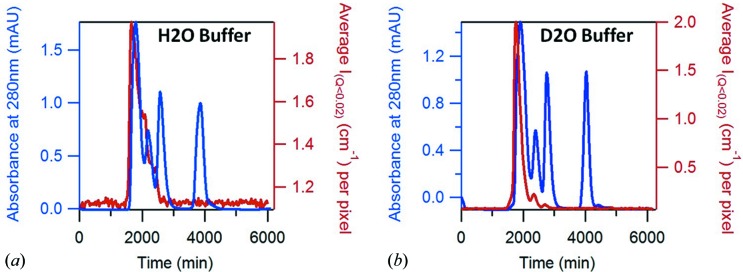
SEC-SANS measurements of SEC calibration standards eluted with 0% (*a*) and 100% (*b*) D_2_O buffer. The absorbance of the sample at 280 nm is plotted in blue, and the averaged SANS intensity between *Q* = 0.007 Å^−1^ and *Q* = 0.02 Å^−1^ is in red. The column (Superdex 75) efficiently separates proteins of molecular weight (MW) from 3000 to 70000 Da.

Size exclusion chromatography standards for calibration were used to test and calibrate the *in situ* chromatography system on D22 while measuring the SANS signal in the 8 m configuration. One 18 mg vial of protein, resuspended in 0.5 ml of buffer, was used per gel filtration, leading to a total concentration of 36 mg ml^−1^. The averaged absolute intensity at a *Q* range of 0.007 ≤ *Q* ≤ 0.02 Å^−1^ is plotted as a function of time in Fig. 2 ▸, together with the UV absorbance at 280 nm, which was measured at a point between the SANS measurement cell and the fraction collector. This experiment was repeated twice: once by eluting the sample with an H_2_O buffer (Fig. 2 ▸ *a*) and once with a D_2_O buffer (Fig. 2 ▸ *b*). The columns enabled good separation of ovalbumin (44 kDa) and myoglobin (17 kDa), while thyroglobulin and γ-globulin eluted in the excluded volume (first peak). Vitamin B12 was visible on the UV absorbance plot but the signal was too small to be analyzed by SANS.

The stagger of these plots reflects the time taken for the sample to travel from the SANS measurement cell to the UV absorbance measurement cell and has to be accounted for in concentration normalization. The UV absorbance profile shows that the separated proteins do not re-mix significantly following their passage through the relatively large SANS measurement cell. Even protonated protein in an H_2_O buffer (the worst combination in terms of the contribution to data from hydrogen incoherent scattering) can be successfully subjected to a SEC-SANS analysis. Data recorded during the gel filtration in D_2_O buffer were submitted to a Guinier analysis using the *GRASP* software, and the results are compared with literature data in Table 1 ▸. Three exposures of 30 s each, taken symmetrically from the top of the peak, were averaged to build the myoglobin SANS curve. Five were taken for ovalbumin and ten for the buffer. The corresponding values of absorbance at 280 nm, extracted from the chromatogram, were averaged for the estimation of concentration using the Beer–Lambert method. The experimental values of *R*_g_ are consistent with those in the literature for both proteins (Goldenberg & Argyle, 2014 ▸; Fujisawa & Kato, 2002 ▸; Ianeselli *et al.*, 2010*a* ▸,*b* ▸; Doster *et al.*, 2003 ▸).

### 3.2. Feasibility: example of a classical case   

**Figure 3:**
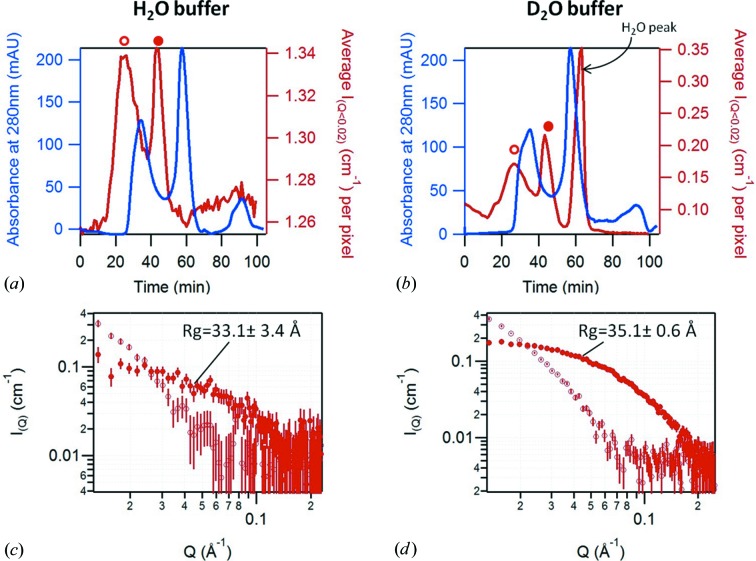
Results of the SEC-SANS measurement of protonated Alba3 in 0% (*a*) and 100% (*b*) D_2_O buffer, showing the absorbance of the sample at 280 nm in blue and the averaged SANS intensity between *Q* = 0.012 Å^−1^ and *Q* = 0.041 Å^−1^ in red. (*c*) and (*d*) show individual SANS curves (30 s exposures) recorded during elution in 0 and 100% D_2_O buffer, respectively, from the top of the first (open circles) and second (full circles) peaks. The *R*_g_ values are obtained using the AutoRg function from *primus* (Konarev *et al.*, 2003 ▸).

Alba3 is a small aggregation-prone protein of 13 kDa. It is used here in its protonated form and at a concentration of 0.9 mg ml^−1^ to represent a typical case for Bio­SANS experiments. The protein was stocked in an H_2_O buffer and eluted either with an H_2_O buffer or a D_2_O buffer. Fig. 3 ▸ shows the two resulting elution profiles as well as the individual SANS curves (30 s exposure) taken from the top of the two elution peaks. As above, the delay between the SANS intensity peak and the UV absorbance peak corresponds to the travel of the protein between the two measurement cells. The UV absorbance, by elution with both buffers, shows three peaks: the first corresponds to some aggregates, the second to the Alba3 monomer, and the third, low-intensity one probably corresponds to traces of imidazole used in the purification protocol that do not produce any coherent neutron scattering owing to the small size of the molecule and the low concentration. The SANS intensity profile obtained by elution with H_2_O buffer shows two peaks; by contrast, elution with D_2_O buffer results in a third peak of high intensity. This peak, which the SANS curve reveals to be essentially incoherent scattering, is attributed to the H_2_O buffer injected with the protein and eluting at the very end of the column. The separation of this peak suggests an efficient buffer exchange. The plots of the individual SANS curves (30 s exposures) illustrate the data quality obtained in each case. As expected, the quality of the data is much better in the case of elution with D_2_O buffer. However, interestingly, even data recorded at low contrast (elution with H_2_O buffer) and low concentration are suitable for Guinier analysis, showing that SEC-SANS is feasible with rather diluted samples.

### 3.3. Specific benefits: example of a sensitive case   

SANS studies are very sensitive to the presence of aggregates or oligomers in the sample. Moreover, the *ab initio* modeling of the molecule in question is only valid on the basis of sample monodispersity and an absence of inter-particle interactions (attractive or repulsive). Consequently, the preparation of a sample optimized for SANS is a real challenge: the intrinsic instability of the molecule is often the main obstacle for a viable analysis using SAS techniques (X-rays or neutrons).

**Figure 4:**
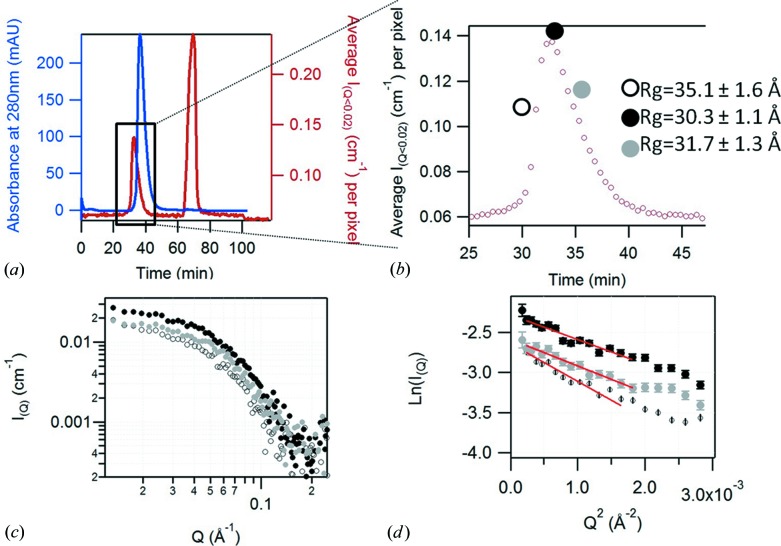
(*a*) Results of the SEC-SANS measurement of protonated Sir2a (4 mg ml^−1^) in 100% D_2_O buffer, showing the absorbance of the sample at 280 nm (in blue) and the averaged SANS intensity between *Q* = 0.012 Å^−1^ and *Q* = 0.041 Å^−1^ (in red). (*b*) Magnified plot of SANS intensity of the Sir2A elution peak. The large symbols show the three individual positions, selected at the beginning (open circle, *R*_g_ = 35.1 ± 1.6 Å), the top (black circle, *R*_g_ = 30.3 ± 1.1 Å) and the end (gray circle, *R*_g_ = 31.7 ± 1.3 Å) of the elution peak, from which are extracted individual SANS curves (*c*) (30 s exposure) and the Guinier plot (*d*) (the red lines being the fits of the Guinier region).

Sir2a is a very delicate protein and its measurement by SANS using a conventional experimental setup led to unsatisfactory results, with clear evidence of aggregation from a Guinier analysis – even when the protein was measured immediately (*i.e.* minutes) after normal SEC purification. This protein therefore provided an excellent test of the SEC-SANS system. Its elution profile is shown in Fig. 4 ▸. Figs. 4 ▸(*a*) and 4 ▸(*b*) show an asymmetric elution peak in UV absorbance as well as in the averaged SANS intensity at small angle (0.012 < *Q* < 0.4 Å^−1^). The Guinier plots for the individual SANS curves (Fig. 4 ▸ *c*) selected at the beginning, the top and the end of the elution peak are shown in Fig. 4 ▸(*d*) and demonstrate that the extracted *R*_g_ varies significantly between curves. The asymmetry in the absorbance peak, coupled with the decrease of *R*_g_ along the elution peak and the fact that this *R*_g_ is larger than the value calculated from the crystalline structure (24 Å), is a clear signature that the system is not monodisperse but rather is composed of monomers and oligomers. Ultracentrifugation measurements later confirmed the presence of trimers and monomers. As we know the oligomeric state of all present species, the SANS curve can then be fitted with the contribution of model oligomers using the *FoXS* software (Schneidman-Duhovny *et al.*, 2010 ▸) or *Oligomer* from the *ATSAS* suite (Konarev *et al.*, 2003 ▸) to estimate the volume fraction of each mixture component. Such an analysis will be detailed in a later publication, together with results of analytical ultracentrifugation.

## 4. Conclusion and future perspectives   

The high neutron fluxes available on ILL SANS instruments, combined with a low-background spectrometer (like D22), enable the acquisition of high-quality data with short exposure times. However, to fully exploit this flux to characterize aggregation-prone biomacromolecules in solution, an online size exclusion chromatography capability offers major advantages for the study of difficult or complex systems.

The results clearly demonstrate the feasibility of the SEC-SANS approach and that it brings real benefits to structural studies of aggregation-prone biomolecules that could not be measured in their monomeric state using a conventional experimental arrangement. The Alba3 results show that the SANS signal from the monomer can be separated from that of its aggregates. The Sir2a system was a much more complicated one, existing as an equilibrium between monomeric and dimeric states; this was also successfully analyzed. To our knowledge, this is the first time that such a combination of SEC and SANS has been carried out. It should be noted that care needs to be taken when designing a SEC-SANS experiment: the contrast should be optimized through a judicious choice of the deuteration of either the solvent or the molecule of interest, or a combination of both. A sufficient concentration must be used (at least 1 mg ml^−1^ for medium sized proteins), but the molecules can be stored in an H_2_O buffer up until the time of measurement since the SEC column is an efficient means of buffer exchange. This property also enables re-use of the same sample for multiple contrast measurements, saving samples and freeing the interpretation from the consequences of sample variability. In the case of a mixed sample consisting of an equilibrium either between two oligomerization states or between two configurations, the SEC-SANS approach cannot separate the different states, but it can provide an aggregate-free averaged SANS curve, enabling an accurate estimation of the relative amount of each state and hence modeling through a structural ensemble approach (Bernadó *et al.*, 2007 ▸; Schneidman-Duhovny *et al.*, 2010 ▸).

The volume probed by the neutron beam, the exposure time and the sample flow rate (70 µl, 30 s and 300 µl min^−1^, respectively, in this study) are the parameters defining the resolution of the SEC-SANS approach and can be tailored to a given study. Although the system is well matched to the study of even rather challenging protein systems, the possibility of very fast aggregation occurring between the exit from the SEC column and the exit from the SANS measurement cell cannot be ruled out.

Following this proof of concept, a dedicated chromatography system, integrated with the instrument hardware and control software, will be implemented in order to tackle new ‘sensitive’ protein structures. Full integration of SEC with the instrument data acquisition will enable us to reduce the flow rate when the protein reaches the scattering measurement cell in order to achieve better statistics (if needed) without affecting the concentration and possible aggregation. In addition, data recorded in streaming mode through the entire sequence will allow finer and better data post processing.

## Figures and Tables

**Table 1:** Experimental (fitted using *GRASP*) structural parameters derived from the results of *in situ* SEC-SANS analysis of the BioRAD calibration standard in 100% D_2_O buffer MW: molecular weight; Extinc. coef.: extinction coefficient; Abs.: absorbance; *I*_(*Q*=0)_: scattering intensity extrapolated at *Q* = 0; *R*_g_: radius of gyration. For comparison, literature data are extracted from references 1: Ianeselli *et al.* (2010*a* ▸,*b* ▸); 2: Doster *et al.* (2003 ▸); 3: Goldenberg & Argyle (2014 ▸); 4: Fujisawa & Kato (2002 ▸).

	Concentration determination			Experimental values (*GRASP*)		Literature data
Parameter	MW (g mol^−1^)	Extinc. coef. [(cm mg ml^−1^)^−1^]	Abs. 280 nm (a.u.)	*I*_(*Q*=0)_ (cm^−1^)	*R*_g_ (Å)	*R*_g_ (Å)
Ovalbumin	44000	0.7	0.272	0.142 ± 0.001	24.0 ± 0.4	23–24^1^
Myoglobin	17000	0.82	0.433	0.0401 ± 0.0005	14.0 ± 0.4	14.8 ± 0.2^2^
						13.9^3^
						17.5 ± 0.1^4^
